# Comparison of bibliometrics for predoctoral Translational Science Training (TST) TL1 Program participants and nonparticipants, male and female participants, and participants from underrepresented and well-represented backgrounds

**DOI:** 10.1017/cts.2022.508

**Published:** 2022-12-01

**Authors:** Christopher R. Frei, Yong-Hee P. Chun, Linda M. McManus, Julie Barker, Amanda M. Moore

**Affiliations:** 1 College of Pharmacy, The University of Texas at Austin, San Antonio, TX, USA; 2 Long School of Medicine, University of Texas Health San Antonio, San Antonio, TX, USA; 3 South Texas Veterans Health Care System, San Antonio, TX, USA; 4 School of Dentistry, University of Texas Health San Antonio, San Antonio, TX, USA

**Keywords:** Translational science, TL1, T32, NIH training program, bibliometrics, disparities, sex differences, underrepresented backgrounds, comparative-effectiveness

## Abstract

Research education and training in Translational Science develops and sustains a workforce to efficiently advance studies designed to improve human health. We evaluated the effectiveness of a Translational Science Training (TST) TL1 Program. Participants had significantly better publications/year, citations/year, *h-index*, and *m-quotient* than nonparticipants. Female and male participants, and participants from underrepresented and well-represented backgrounds, performed similarly on all bibliometric assessments. Finally, TST/TL1 Program participants outperformed students from other PhD programs at our institution. This analysis suggests that the TST/TL1 Program has been effective for participants, including those who are female and from underrepresented backgrounds.

## Background and Introduction

Research education and training in Translational Science (TS) develops and sustains a workforce to efficiently advance clinical and translational studies designed to improve human health. To meet this strategic goal, we operate a Translational Science Training (TST) TL1 Program (TL1 TR002647), a component within our Institute for the Integration of Medicine and Science (IIMS), which is home to our Clinical and Translational Science Award (CTSA) (UL1 TR002645) and serves the communities of Central and South Texas.

The TST/TL1 Program was initiated in 2009, concurrent with our first CTSA. Initial support (2009–2013) was provided by a 5-year grant from the University of Texas System with matching funds from the University of Texas Health Science Center San Antonio (UTHSCSA). Thereafter (2013–2023), support was received from the National Institutes of Health (NIH) through a CTSA-related National Research Service Award (NRSA) provided by the National Center for Advancing Translational Sciences (NCATS).

Trainees were selected annually using an NIH style, comprehensive, expert panel review of applications. Successful applicants received 1 year awards that included stipends plus research and travel support. Initially, the Program only supported predoctoral trainees, but in 2018, the Program was expanded to include both predoctoral and postdoctoral Trainees. This analysis is limited to those 66 predoctoral trainees who have completed TST/TL1 training so far.

Since inception, the Program has enhanced the quality and scope of predoctoral research training through interdisciplinary Clinical and Translational Science (CTS) training and mentorship. Trainees have used our framework to construct individualized research training and career development programs that facilitated their capacity to move science into human studies and in the community.

The primary activity of the TST/TL1 Trainee is to conduct research within the ongoing investigative program of an experienced scientist. The TST/TL1 Program oversees the mentoring process to ensure a supportive and effective mentoring relationship. Trainees must have a committed mentor when they apply to the Program. The Program takes steps to ensure mentor quality. Trainees who have Assistant Professors as mentors must also have more experienced comentors. All mentors are encouraged to complete training that introduces effective strategies for mentoring. Trainees are also offered training to help them better understand their role in effective mentoring. Trainees complete an Individual Development Plan (IDP) within 3 months of their appointment to the TST Program. Alignment of Mentor and Trainee expectations is documented in a required Compact, which is based on the AAMC Compact between Biomedical Graduate Students and Their Research Advisors [1].

Trainees engage in required TS coursework that describes TS investigative efforts and unresolved hypotheses in a format that facilitates case-based discussions and debate. Course directors are successful TS investigators from medicine, nursing, dentistry, and pharmacy. Course content focuses on topics such as Translational Science, Responsible Conduct, Research Design and Analysis, Team Science and Leadership, Cultural Proficiency, Scientific Communication, Business of TS, and Evidence-Based Implementation and Policy.

TST/TL1 programmatic activities, including TS coursework, ensure that Trainees recognize the relationship between applied TS and contemporary biomedical research. Monthly TST/TL1 Program Meetings reinforce TS concepts, community engagement, dissemination and implementation (D&I), diversity, equity, inclusion, and accessibility (DEIA), career development, mentoring, and wellness. Together with CTSA KL2 Scholars, TST/TL1 Trainees participate in a TS Roundtable that integrates concepts in process innovation, systems thinking, and boundary crossing, along with team science and cultural diversity. Trainees also participate in research laboratory activities/meetings, seminars/conferences/workshops, scientific meetings, and manuscript and grant preparation workshops.

TST/TL1 Trainees are required to participate in a TS practicum course. This experiential learning course immerses Trainees in activities relevant to their primary research and encourages them to move their work along the TS spectrum. Over 6 months, Trainees undergo customized experiential activities like shadowing a clinician, assisting in a clinical trial, and exploring types of research that are new to them. The TS Practicum is highly individualized for each person, and Trainees are encouraged to include a community-engagement activity in their practicum.

Thus, the Trainees gained awareness for scientific and cultural challenges in the translation of basic science observations and the interdisciplinary teams needed to support the continuum of TS for benefit in patient care, community, economy, and policy. The ‘phenotype’ of predoctoral Trainees at IIMS are trainees who are prepared for an impactful career as translational scientist or clinician-translational scientist.

The primary objective of this analysis was to compare the bibliometrics of TST/TL1 Program participants and nonparticipants. Secondary objectives included comparisons of Program participants by sex and underrepresented status and comparisons of Program participants to students in other UTHSCSA PhD programs at our institution.

## Methods

Bibliometric data were gathered using Scopus and included: publications per year, citations per year, coauthors per year, *h-index*, and *m-quotient*. Numerical data were assessed for normality. Since they were not normally distributed, the Wilcoxon Rank Sum test was used to compare numerical data. Nominal data (sex and underrepresented status) were compared using the chi-square test, unless the expected counts were less than 5 in any cell; in which case, the Fisher’s exact test was used instead of the chi-square test. P-values less than a pre-specified alpha level of 0.05 were statistically significant.

## Results

Among the 66 predoctoral TST/TL1 Trainees, 65% (43/66) were women and 29% (19/66) were underrepresented (UR) in science. Trainees produced an average of eight publications each since starting the Program and one-third subsequently received independent training awards (AHA, AACR, AFAR, NIH NRSA, F99/K00, K08, K99/R00).

Those predoctoral students who applied and were accepted for the TST/TL1 Program (participants) outperformed those who applied but were not selected for the Program (non-participants). Participants had significantly better publications per year, citations per year, *h-index*, and *m-quotient* (*m-quotient* = *h-index* divided by years since first publication) (Table 1). Importantly, female and male participants, and participants from underrepresented and well-represented backgrounds, performed similarly on all bibliometric assessments (Table 2).

**Table 1.** Comparison of characteristics and bibliometrics for TST/TL1 participants and nonparticipants

Characteristic	Participants (n = 66)	Non-participants (n = 62)	P-value
Female, %	65%	58%	0.68
Underrepresented, %	29%	24%	0.56
Publications per year, median (IQR)	0.7 (0.4–1.2)	0.5 (0.0–1.0)	*0.04*
Citations per year, median (IQR)	15.3 (3.5–27.6)	5.2 (0.0–19.1)	<0.01
Coauthors per year, median (IQR)	3.0 (1.4–6.0)	1.6 (0–4.9)	0.06
*H-index*, median (IQR)	4.5 (2.0–8.0)	2.0 (0.0–6.0)	<0.01
*M-quotient*, median (IQR)	0.5 (0.3–0.7)	0.3 (0.0–0.6)	0.01

Nonparticipants = TST/TL1 applicants were those who were not selected for participation in the Program.

IQR = interquartile range.

*M-quotient* = *h-index* divided by years since first publication.

**Table 2.** Comparison of bibliometrics for TST/TL1 participants by sex and underrepresented status

Characteristic, median (IQR)	Female participants (n = 43)	Male participants (n = 23)	P-value
Publications per year	0.8 (0.5–1.2)	0.5 (0.3–1.1)	0.25
Citations per year	18.0 (3.8–29.0)	6.6 (2.6–24.9)	0.27
Coauthors per year	3.0 (1.5–6.8)	2.3 (1.3–4.7)	0.30
*H-index*	5.0 (2.0–8.0)	4.0 (2.0–7.0)	0.40
*M-quotient*	0.6 (0.4–0.8)	0.4 (0.3–0.7)	0.16
Characteristic, median (IQR)	Underrepresented participants (n = 19)	Well-represented participants (n = 47)	P-value
Publications per year	0.7 (0.5–1.0)	0.8 (0.4–1.2)	0.58
Citations per year	8.8 (2.6–36.8)	16.7 (4.3–25.7)	0.66
Coauthors per year	3.8 (1.8–6.8)	2.4 (1.4–5.8)	0.54
*H-index*	3.0 (1.0–8.0)	5.0 (3.0–8.0)	0.21
*M-quotient*	0.5 (0.3–0.7)	0.6 (0.3–0.8)	0.58

IQR = interquartile range.

*M-quotient* = *h-index* divided by years since first publication.

TST/TL1 participants also outperformed predoctoral students from our institution’s Integrated Biomedical Sciences (IBMS) PhD Program, MD–PhD Program, and TS PhD Program (a standalone program jointly offered by IIMS through four partnering universities that is independent from the TST/TL1 Program) (Table 3). TST/TL1 participants had significantly more citations per year and higher *h-index* than our IBMS students. TST/TL1 participants also had significantly more publications per year, citations per year, coauthors per year (possibly indicating more collaboration and teamwork), and higher *h-index* and *m-quotient* than our MD–PhD students. Finally, TST/TL1 participants also had significantly more citations per year than our TS PhD students.

**Table 3.** Comparison of bibliometrics for TST/TL1 participants and other predoctoral programs at our institution

Characteristic, median (IQR)	TST/TL1 participants (n = 66)	IBMS students (n = 39)	P-value	MD-PhD students (n = 57)	P-value	TS PhD students (n = 48)	P-value
Publications per year	0.7 (0.4–1.2)	0.8 (0.4–1.3)	0.73	0.4 (0.0–1.1)	*0.02*	0.7 (0.0–1.4)	0.80
Citations per year	15.3 (3.5–27.6)	6.3 (1.0–15.3)	*0.02*	4.6 (0.0–15.5)	*0.01*	5.1 (0.0–20.6)	*0.04*
Coauthors per year	3.0 (1.4–6.0)	3.4 (1.4–7.5)	0.69	0.9 (0.0–6.0)	*0.02*	2.6 (0.0–6.0)	0.34
*H-index*	4.5 (2.0–8.0)	2.0 (2.0–4.0)	*<0.01*	2.0 (0.0–5.0)	*<0.01*	2.0 (0.0–8.0)	0.14
*M-quotient*	0.5 (0.3–0.7)	0.5 (0.3–0.7)	0.92	0.3 (0.0–0.61)	*0.01*	0.3 (0.0–0.6)	0.06

P-values are for the comparison of each PhD Program to the TST/TL1 Program (reference group).

IQR = interquartile range; IBMS = Integrated Biomedical Sciences PhD Program; TS PhD = Translational Sciences PhD Program.

*M-quotient* = *h-index* divided by years since first publication.

## Discussion and Conclusion

TST/TL1 participants outperformed nonparticipants and students from our institution’s IBMS Program, MD–PhD Program, and TS PhD Program on a battery of bibliometric assessments. Moreover, among TST/TL1 participants, males and females, and participants from underrepresented and well-represented backgrounds, performed similarly on all bibliometric assessments. This analysis suggests that the TST/TL1 Program has been beneficial for participants. Furthermore, the Program has been able to achieve similar bibliometric outcomes in men and women and in persons from underrepresented and well-represented backgrounds, which suggests that the Program’s approach to training those from backgrounds underrepresented in science is effective.

The study has limitations, including possible selection bias, as participants for the TST/TL1 Program are selected by a rigorous NIH-style review panel and those with the best scores are admitted to the Program. Furthermore, the study did not systematically evaluate contextual factors, like student history, student maturation, scientific discipline, authorship patterns, and type of employment post-Program, which might mediate the findings.

Nevertheless, it is encouraging that the TST/TL1 Program fared well when compared to other prominent PhD programs at our institution, and that participants from backgrounds underrepresented in science benefitted to a similar extent as those from well-represented backgrounds.
